# Growth Curves for Children with X-linked Hypophosphatemia

**DOI:** 10.1210/clinem/dgaa495

**Published:** 2020-07-28

**Authors:** Meng Mao, Thomas O Carpenter, Michael P Whyte, Alison Skrinar, Chao-Yin Chen, Javier San Martin, Alan D Rogol

**Affiliations:** 1 UBiometrics, Ultragenyx Pharmaceutical Inc., Novato, California; 2 Department of Pediatrics, Department of Orthopaedics and Rehabilitation, Yale University School of Medicine, New Haven, Connecticut; 3 Division of Bone and Mineral Diseases, Department of Internal Medicine, Washington University School of Medicine, St. Louis, Missouri; 4 Center for Metabolic Bone Disease and Molecular Research, Shriners Hospitals for Children - St Louis, St Louis, Missouri; 5 Pediatrics, University of Virginia School of Medicine, Charlottesville, Virginia; 6 Clinical Outcomes, Research and Evaluation, Ultragenyx Pharmaceutical Inc., Novato, California; 7 Clinical Development, Ultragenyx Pharmaceutical Inc., Novato, California

**Keywords:** X-linked hypophosphatemia, FGF23, PHEX, growth curve, rickets

## Abstract

**Context:**

We characterized linear growth in infants and children with X-linked hypophosphatemia (XLH).

**Objective:**

Provide linear growth curves for children with XLH from birth to early adolescence.

**Design:**

Data from 4 prior studies of XLH were pooled to construct growth curves. UX023-CL002 was an observational, retrospective chart review. Pretreatment data were collected from 3 interventional trials: two phase 2 trials (UX023-CL201, UX023-CL205) and a phase 3 trial (UX023-CL301).

**Setting:**

Medical centers with expertise in treating XLH.

**Patients:**

Children with XLH, 1-14 years of age.

**Intervention:**

None.

**Main Outcome Measure:**

Height-for-age linear growth curves, including values for the 5^th^, 10^th^, 25^th^, 50^th^, 75^th^, 90^th^, and 95^th^ percentiles for children with XLH compared to population norms.

**Results:**

A total of 228 patients (132 girls, 96 boys) with 2381 height measurements were included. Nearly all subjects (> 99%) reported prior management with supplementation therapy. Compared to the Center for Disease Control and Prevention growth curves, boys at age 3 months, 6 months, 9 months, 1 year, and 2 years had median height percentiles of 46%, 37%, 26%, 18%, and 5%, respectively; for girls the median height percentiles were 52%, 37%, 25%, 18%, and 7%, respectively. Annual growth in children with XLH fell below that of healthy children near 1 year of age and progressively declined during early childhood, with all median height percentiles < 8% between 2 and 12 years old.

**Conclusion:**

Children with XLH show decreased height gain by 1 year of age and remain below population norms thereafter. These data will help evaluate therapeutic interventions on linear growth for pediatric XLH.

X-linked hypophosphatemia (XLH; OMIM #307800) is a rare disorder featuring renal phosphate wasting and is considered the most common heritable form of rickets and osteomalacia (1, 2). Caused by loss-of-function mutations in the *PHEX* (phosphate-regulating endopeptidase homolog, X-linked) gene, XLH is characterized by high circulating levels of fibroblast growth factor 23 (FGF23). Excess FGF23 leads to hypophosphatemia and inappropriately low circulating 1,25 dihydroxyvitamin D (1,25[OH]_2_D), with consequent skeletal deformities and growth impairment.

While impaired growth is a hallmark of XLH, limited literature characterizes the course of growth, particularly when deceleration of height velocity occurs. An observational study of 127 untreated pediatric patients with XLH showed that normal length/height is observed at birth, but growth impairment develops during infancy/early childhood (3).

Two studies examined a smaller number of patients with XLH (< 20) whose signs and symptoms of their disease were managed with supplementation therapy, consisting of multiple daily doses of oral phosphate salts and 1 or more daily doses of active vitamin D (3, 4). Children with XLH have typically been managed with this therapy since the 1980s, individually titrated, and frequently monitored to minimize the risk of nephrocalcinosis and hyperparathyroidism (1, 2, 5). Supplementation therapy demonstrated limited increases in growth or height standard deviation scores, with a greater impact when initiated at an early age (2–4). Other small studies report a limited benefit from adding growth hormone therapy to this regimen (6, 7).

Burosumab, a fully human monoclonal antibody against FGF23, was approved for the treatment of XLH in the United States, Europe, and Canada in 2018 (conditions of approval vary) (8). In phase 2 and 3 pediatric trials, treatment with burosumab resulted in modest, but significant, increases in standing height z-scores and improved lower limb deformities, which contribute to growth impairment (9–11).

To better characterize growth impairment in patients with XLH, we constructed height growth curves for affected children, the vast majority of whom were receiving supplementation therapy, from birth to early adolescence (or until enrollment in clinical trials). Our findings provide an important reference to assess treatments on growth outcomes in XLH.

## Materials and Methods

**Figure 1.**
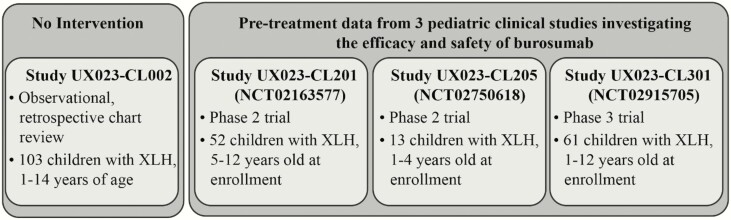
Pooled datasets for XLH growth curves. Abbreviation: XLH, X-linked hypophosphatemia.

Growth data from 4 prior XLH studies were pooled to construct growth curves (Fig. 1). UX023-CL002 was an observational, retrospective chart review of 103 children with XLH, 1–14 years of age. The remaining 3 studies were interventional clinical trials that investigated the efficacy and safety of burosumab for the treatment of children with XLH. However, only historical, preburosumab growth data collected from these clinical trials were used for this analysis. That is, no data in this analysis represent children receiving current or previous treatment with burosumab, though nearly all were receiving supplementation therapy. The 3 interventional burosumab clinical studies included a phase 2 trial (UX023-CL201, NCT02163577) in 52 children with XLH, ages 5–12 years old at enrollment (11); a phase 2 trial (UX023-CL205, NCT02750618) in 13 children with XLH, 1–4 years old at enrollment (9); and a phase 3 trial (UX023-CL301, NCT02915705) in 61 children with XLH, 1–12 years old at enrollment (10).

Eligibility criteria for these clinical trials were similar but varied slightly across studies, as described below. The complete list of inclusion and exclusion criteria are available in the full protocols published as supplementary material together with the study’s primary results for studies UX023-CL201, UX023-CL205, and UX023-CL301 (9–11). All studies required a diagnosis of XLH based on the presence of a *PHEX* mutation in the patient or directly related family member, with appropriate inheritance; studies UX023-CL201 and UX023-CL002 also permitted a diagnosis of XLH based upon an appropriate biochemical profile. For study UX023-CL002, the only additional key criteria was willingness to provide access to medical records and radiographic images from at least 2 timepoints between 5 and 14 years of age, inclusive.

The 3 interventional studies each required low levels of fasting serum phosphorus (≤ 0.90 mmol/L or ≤ 2.8 mg/dL for study UX023-CL201; ≤ 0.97 mmol/L or ≤ 3.0 mg/dL for studies UX023-CL205 and UX023-CL301) and serum creatinine levels within the age-appropriate normal range. Study UX023-CL301 also required a fasting serum 1,25(OH)_2_D level ≥ 38 pmol/L or ≥ 16 pg/mL.

Eligibility criteria for all 3 interventional studies also included evidence of radiographic rickets at screening. Importantly, because this requirement was assessed during screening, the severity of bone disease is less certain at the time that historical pretreatment growth data were collected (ie, prescreening). The initial 36 subjects in Study UX023-CL201 were required to have radiographic evidence of bone disease, including rickets at the wrist or knee and/or femoral/tibial bowing; the last 16 subjects to enroll, due to a protocol amendment, were required to have a baseline Thacher Rickets Severity Knee Score ≥ 1.5. For study UX023-CL301, subjects were required to have a baseline Thacher Rickets Severity Total Score ≥ 2.0. For study UX023-CL205, at least 5 of the enrolled subjects were required to have a baseline Thacher Rickets Severity Knee Score ≥ 1.5. Subjects in studies UX023-CL201 and UX023-CL301 were also required to have a standing height ≤ 50^th^ percentile for age- and gender-matched local norms to enroll. Certain medications, such as growth hormone, were restricted for a transient period of time leading up to enrollment to participate in the clinical trial, but this time frame does not necessarily represent the time frame during which historical, preburosumab treatment growth data were collected.

### Statistical analysis

We constructed height-for-age growth curves for children with XLH, and specific line curves were generated to represent values for the 5^th^, 10^th^, 25^th^, 50^th^, 75^th^, 90^th^, and 95^th^ percentiles for these children. Separate curves were generated for boys and girls. These XLH height-for-age growth curves were then compared to growth curves representing population norms from the Center for Disease Control and Prevention (CDC; year 2000). The Lambda-Mu-Sigma (LMS) function (a function to fit LMS curves for centile estimation) in R package Generalised Additive Models for Location Scale and Shape (GAMLSS) was used to construct the XLH growth curves. To smoothly transition from recumbent length to standing height, 0.8 cm was subtracted from recumbent length values before pooling the data with standing height per established methodology from Kuczmarski et al (12). Data are expressed as group 50^th^ percentile (median) for boys and girls separately as height in centimeters, z-score, percentile, and as annual height increment in centimeters. To statistically compare the growth of children with XLH versus the normal population and growth in boys versus girls over time, height z-scores of children with XLH were compared to median height of the normal population (z-score = 0) and between boys and girls by 2-tailed t-test at ages 0, 3 months, 6 months, 9 months, 1 year, and every year up to 13 years. When multiple measurements were available for a specific age, the measurement closest to the given age timepoint but prior to the subsequent timepoint was selected for the analysis. All supplemental materials and figures are located in the Dryad digital research materials repository (13).

## Results

A total of 2381 height measurements from 228 subjects (132 girls, 96 boys) were analyzed from the 4 studies, except 1 subject from the phase 3 trial who lacked pretreatment growth data (Table 1). The number of subjects in each geographic region was 132 in the United States, 51 in Europe, 29 in Canada, 9 in Australia, and 7 in Asia. Most subjects (72%, 165/228) had confirmed *PHEX* mutations. The remainder were confirmed by a family history of *PHEX* mutations and biochemical profile. Nearly all subjects (99%; 225/229) reported prior management with oral phosphates and active vitamin D metabolites. Mean (SD) RSS was 2.3 (1.34) across the studies (baseline RSS was used for UX023-CL201, UX023-CL205, and UX025-CL202; the last X-ray collected was used for CL002, the observational study). The lowest RSS was seen in UX023-CL002 and the highest was seen in UX023-CL301 (Table 1). On average, subjects contributed 10 data points to this analysis.

**Table 1.** Subject characteristics collected at the initiation of each study

	UX023-CL002	UX023-CL201	UX023-CL205	UX023-CL301	
Assessment	(N = 103)	(N = 52)	(N = 13)	(N = 61)	Total
Age of historical data, years, min–max	1–14	0–12	0–4	0–12	0–14
Number of subjects included from the study, n	103	52	13	60^*a*^	228
Female, n (%)	67 (65)	28 (54)	4 (31)	33 (55)	132 (58)
White, n (%)	71 (69)	46 (89)	12 (92)	49 (82)	178 (78)
Number of subjects who received prior supplementation therapy, n (%)	102 (99)	50 (96)	13 (100)	60 (100)	225 (99)
Age at initiation of supplementation therapy, years, mean (SD)	2.5 (2.2)	2.1 (1.3)	1.6 (1.5)	2.3 (2.3)	2.3 (2.1)
Birthday range	1970–2012	2002–2010	2011–2015	2004–2016	1970–2016
RSS total score, mean (SD)^*b*^	1.2 (0.88)c	1.8 (1.09)	2.9 (1.37)	3.2 (1.06)	2.3 (1.34)

Abbreviations: max, maximum; min, minimum; SD, standard deviation.

^*a*^One subject lacked prestudy growth data.

^*b*^Baseline data were used for CL201, CL205, and CL301. For CL002, the last measurement collected from the patient’s medical record was used.

^*c*^Only the subgroup of patients with radiographs was included, n = 60.

**Figure 2.**
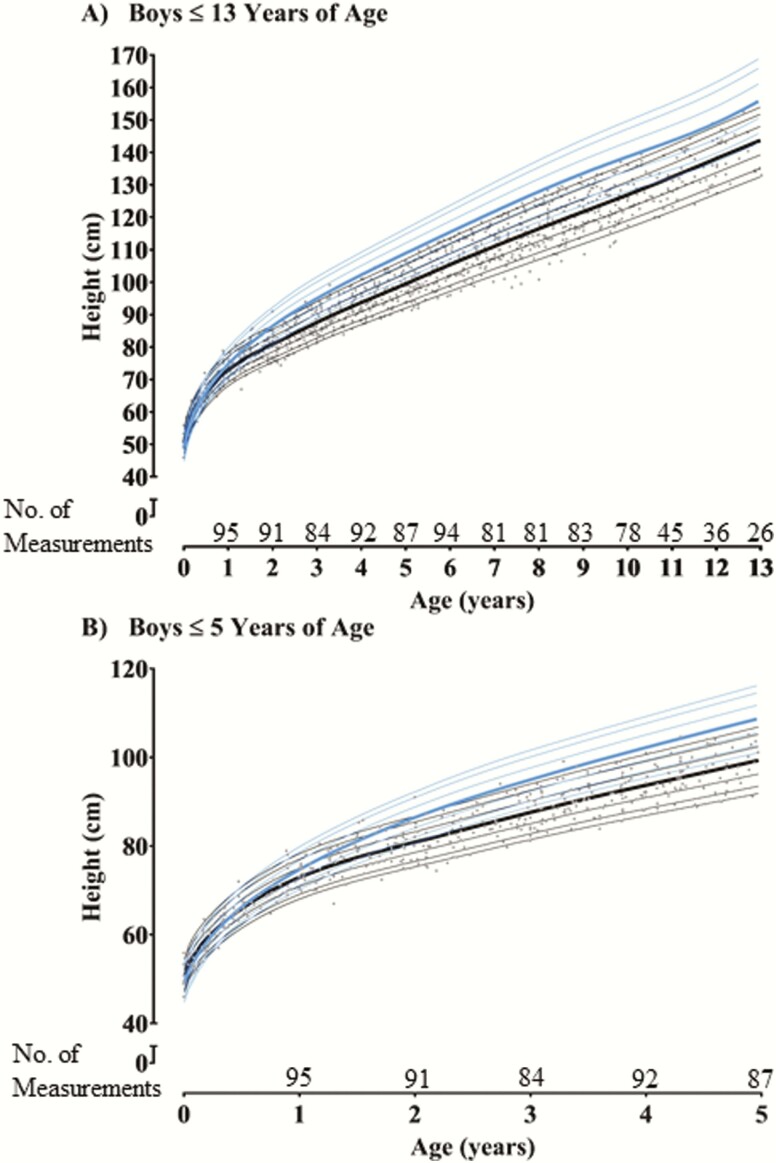
X-linked hypophosphatemia (XLH) and Center for Disease Control and Prevention (CDC) growth curves for boys. **A:** Boys ≤ 13 years of age. **B:** Boys ≤ 5 years of age. CDC growth curve lines (year 2000) for boys in blue, with the 50^th^ percentile bolded. XLH growth curve lines in black: 5^th^, 10^th^, 25^th^, 50^th^ (bolded), 75^th^, 90^th^, and 95^th^ percentile generated from prior studies in the burosumab program before treatment with burosumab (Studies UX023-CL002, UX023-CL201, UX023-CL205, UX023-CL301). Grey dots (●) indicate individual XLH data points before treatment with burosumab. To smoothly transition from recumbent length to standing height, 0.8 cm was subtracted from recumbent length values before pooling data with standing height per established methodology from Kuczmarski et al (12).

**Figure 3.**
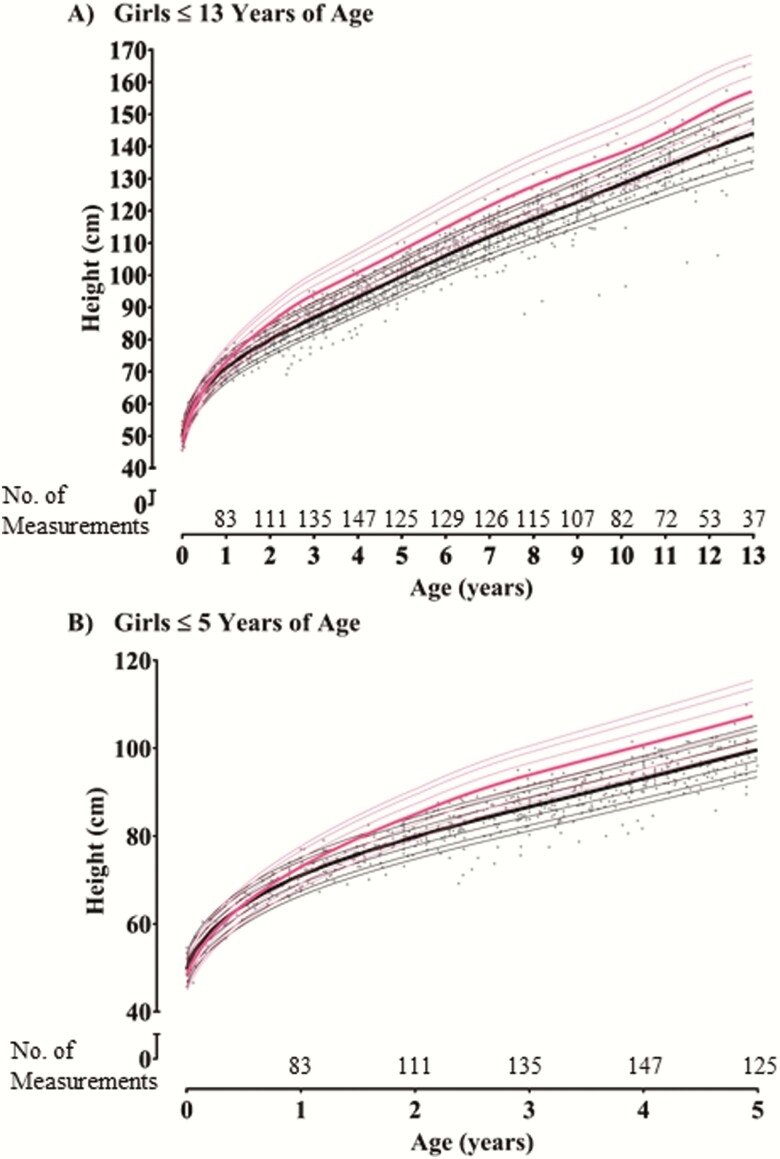
X-linked hypophosphatemia (XLH) and Center for Disease Control and Prevention (CDC) growth curves for girls. **A:** Girls ≤ 13 years of age. **B:** Girls ≤ 5 years of age. CDC growth curve lines (year 2000) for girls in pink, with the 50^th^ percentile bolded. XLH growth curve lines in black: 5^th^, 10^th^, 25^th^, 50^th^ (bolded), 75^th^, 90^th^, and 95^th^ percentile generated from prior studies in the burosumab program before treatment with burosumab (Studies UX023-CL002, UX023-CL201, UX023-CL205, UX023-CL301). Grey dots (●) indicate individual XLH data points before treatment with burosumab. To smoothly transition from recumbent length to standing height, 0.8 cm was subtracted from recumbent length values before pooling data with standing height per established methodology from Kuczmarski et al (12).

Compared to the CDC growth curves, for boys at 3 months, 6 months, 9 months, 1 year, and 2 years of age the median height percentiles were 46%, 37%, 26%, 18%, and 5% respectively (Fig. 2 and Table 2); for girls the median height percentiles were 52%, 37%, 25%, 18%, and 7%, respectively (Fig. 3 and Table 2). The annual height increment in children with XLH began to deviate from that of healthy children at 6 months of age. Z-scores were significantly different from the median normal population (z-score = 0) at 6 months for both girls and boys (Supplemental Table 1) (13). Height increment progressively declined during early childhood, with all median height percentiles < 8% between 2 and 12 years of age. A similar pattern of decline in growth in children with XLH approaching 1 year of age was observed in both males and females when the data are presented as a median length/height z-score (Table 2**).** In general, height z-scores were numerically lower in boys than girls after 1 year of age; however, these differences were not significant (Supplemental Table 1) (All supplemental materials and figures are located in the Dryad digital research materials repository (13)). XLH-specific growth curves for both boys and girls without the CDC curves and individual XLH data points before burosumab treatment are provided for reference in Supplemental Figs. 1 and 2 (13).

**Table 2.** Median heights (cm, z-score, and percentile based on CDC norms) in children with XLH by age

	Girls			Boys		
Age, years	Height, cm	Corresponding Z-Score	Corresponding Height Percentile	Height, cm	Corresponding Z-Score	Corresponding Height Percentile
**0 mo.**	49.6	0.14	55.4	50.4	0.17	56.8
**3 mo.**	59.4	0.06	52.5	60.6	-0.09	46.4
**6 mo.**	64.4	-0.33	37.2	66.1	-0.34	36.6
**9 mo.**	68.0	-0.69	24.6	69.9	-0.65	25.8
**1**	71.1	-0.93	17.6	72.9	-0.92	18.0
**2**	79.8	-1.48	6.9	80.9	-1.61	5.4
**3**	86.7	-1.87	3.0	87.6	-2.02	2.2
**4**	93.1	-1.82	3.5	93.7	-2.04	2.1
**5**	99.7	-1.75	4.0	99.5	-2.01	2.2
**6**	106.0	-1.78	3.7	105.4	-1.98	2.4
**7**	111.9	-1.84	3.3	111.0	-2.01	2.2
**8**	117.4	-1.83	3.4	116.4	-2.04	2.1
**9**	122.7	-1.70	4.5	121.7	-1.97	2.4
**10**	128.2	-1.49	6.8	126.9	-1.82	3.4
**11**	133.7	-1.42	7.8	132.3	-1.63	5.2
**12**	139.0	-1.63	5.1	138.0	-1.51	6.5
**13**	143.9	-1.89	2.9	143.7	-1.59	5.6

Data are presented as median. Corresponding height percentile is based on CDC norms for age and sex. Abbreviations: CDC, Center for Disease Control and Prevention; mo., months; XLH, X-linked hypophosphatemia.

The annual height increment in centimeters of the XLH 50^th^ percentile growth curve was compared to normal reference ranges (14–16) in Table 3. In the 0–1 age group, the annual height gain for boys with XLH was 21.5 cm compared to 25 cm for healthy population norms; for girls with XLH the value was 22.5 cm compared to 25 cm for healthy population norms. After the 0–1 age group, annual height gain in healthy population norms remained greater than in children with XLH through early adolescence.

**Table 3.** Annual height increment (cm) by age in children with XLH compared to healthy reference range

	Girls			Boys		
Age, years	Normal Reference, cm/year (14–16)	XLH Median Annual Height Increment, cm	Difference (Normal–XLH)	Normal Reference, cm/year (14–16)	XLH Median Annual Height Increment, cm	Difference (Normal–XLH)
**Birth**	50^*a*^	50^*a*^	0	50^*a*^	50^*a*^	0
**0–1**	25	21.5	3.5	25	22.5	2.5
**1–2**	10	8.7	1.3	10	8.0	2
**2–3**	8.6	6.9	1.7	8.3	6.7	1.6
**3–4**	7.6	6.4	1.2	7.4	6.1	1.3
**4–5**	6.8	6.6	0.2	6.8	5.8	1
**5–6**	6.7	6.3	0.4	6.8	5.9	0.9
**6–7**	6.4	5.9	0.5	6.4	5.6	0.8
**7–8**	6.1	5.5	0.6	6.0	5.4	0.6
**8–9**	5.9	5.3	0.6	5.7	5.3	0.4
**9–10**	6.2	5.5	0.7	5.5	5.2	0.3
**10–11**	6.6	5.5	1.1	5.5	5.4	0.1
**11–12**	6.3	5.3	1	6.1	5.7	0.4
**12–13**	4.8	4.9	-0.1	7.1	5.7	1.4

Abbreviation: XLH, X-linked hypophosphatemia.

^*a*^Data are reported for length at birth. All data are presented as median values.

## Discussion

This analysis is the largest dataset (228 children) characterizing growth in children with XLH to date. Results demonstrate that children with XLH are born at average length percentiles and show diminished growth by 1 year of age, regardless of expressing the findings as a length z-score, percentile, or annual height growth. Though nearly all subjects (99%) had received supplementation therapy, usually starting at about 1–2 years of age, their growth progressively declined during early childhood and remained deficient thereafter.

Consistent with previously published smaller studies assessing height in children with XLH (3, 4), there was a noteworthy decline in age- and sex-matched height z-scores/percentiles after 1 year of age in both boys and girls, with the steepest declines observed as early as 9 months old and the lowest height z-scores/percentiles observed after 3 years of age due to a lack of catch-up growth (3, 4, 6). Also consistent with previous research, these findings suggest that treatment may have a greater impact when initiated at an early age.

The data used to construct these growth curves were derived from a retrospective chart review and 3 interventional clinical trials. These interventional trials had inclusion criteria regarding the presence of radiographic rickets and a height requirement (< 50^th^ percentile for age- and sex-matched local norms) at enrollment. Importantly, entry criteria were assessed at the time of screening and do not necessarily reflect height or rickets severity during preburosumab treatment years (ie, prescreening), or more specifically, at the time for which data were collected for this analysis. Additionally, most subjects (64%, 147/228) included in this analysis participated in a study that either did not have enrollment criteria related to rickets severity and height or required evidence of radiographic rickets that was not defined by a rickets scoring system. Our findings are concordant with smaller observational studies examining growth in children with XLH (3, 4, 6). Lastly, these entry criteria reflect the signs and symptoms of the majority of patients with XLH at the ages when enrolled.

Several factors could not be assessed, which may affect growth. Tanner stage was not collected, so the onset of puberty is not accounted for in these growth curves. This is consistent with other commonly used growth curves that do not account for Tanner staging. Patients may undergo surgery to correct bone deficits; however, the small number of patients that received surgical treatment prevent meaningful analysis. Finally, most patients were taking supplementation therapy with vitamin D analogs and oral phosphate, although the degree of adherence to these regimens is unknown. Other limitations to be addressed with future research were that growth curves were only constructed for patients younger than 13 years, and fewer data points were available approaching the age of 13 years. Growth data from older children and adolescents will be addressed in an ongoing XLH Disease Monitoring Program—a prospective, multinational, longitudinal outcomes study to systematically describe the clinical, radiographic, and biochemical manifestations of XLH in children and adults regardless of treatment (NCT03651505). The XLH Disease Monitoring Program will pursue the continued assessment of growth, allowing for the description of potential changes during the adolescent growth spurt, which were not captured in the data presented here.

In conclusion, children with XLH manifest decreased height velocity by 1 year of age. Growth curves generated here from children with XLH, nearly all of whom were receiving supplementation therapy, provide an important reference for objective evaluations of new interventions on growth in children/adolescents with XLH.

## Data Availability

The datasets generated during and/or analyzed during the current study are not publicly available but are available from the corresponding author on reasonable request.

## Additional Information

***Disclosure Summary:*** M.M., C.-Y.C., and J.S.M. were employed at Ultragenyx Pharmaceutical Inc. at the time that the work for this manuscript was completed. M.M. is now an employee of Ascendis Pharmaceuticals, C.-Y.C. is now an employee of 89bio, and J.S.M. is now an employee of Arrowhead Therapeutics. T.O.C. and M.P.W. were clinical investigators for studies sponsored by Ultragenyx Pharmaceutical Inc. in partnership with Kyowa Hakko Kirin Co., Ltd. A.S. is an employee and shareholder of Ultragenyx Pharmaceutical Inc. A.D.R. has served as a consultant for Ultragenyx Pharmaceutical Inc.
